# Wheat husk-based sorbent as an economical solution for removal of oil spills from sea water

**DOI:** 10.1038/s41598-023-29035-8

**Published:** 2023-02-13

**Authors:** Basma M. Omar, Soad A. Abdelgalil, Hala Fakhry, Tamer M. Tamer, Mervat A. El-Sonbati

**Affiliations:** 1 Department of Environmental Sciences, Faculty of Science, Damietta University, Damietta, 34517 Egypt; 2 Bioprocess Development Department, Genetic Engineering and Biotechnology Research Institute (GEBRI), City of Scientific Research and Technological Applications (SRTA-City), Universities and Research Institutes Zone, New Borg El-Arab City, Alexandria, 21934 Egypt; 3 Polymer Materials Research Department, Advanced Technology and New Materials Research Institute (ATNMRI), City of Scientific Research and Technological Applications (SRTA-City), New Borg El-Arab City, Alexandria, 21934 Egypt

**Keywords:** Environmental sciences, Environmental impact

## Abstract

Oil spills are a significant threat to the marine ecosystem that requires immediate removal from the oceanic environment. Many technologies have been employed to clean up oil spills. Of these, adsorption has scored a prominent success due to the high efficiency, economic viability, environmental friendship, and ease of application. The utilization of agricultural waste to produce biosorbents have been considered as an ecofriendly and efficient approach for removing oil. Thus, a new low-cost oil adsorbent was prepared via esterification of the wheat straw (Str) with a hydrophobic benzoyl group, the resulting copolymer (Str-co-Benz) was characterized by FTIR, TGA, DSC, and SEM and used at laboratory scale. The oil spill cleanup process was conducted using a crude oil-natural seawater system under different adsorption conditions such as oil concentration, adsorbent dose, agitation time and speed. Equilibrium studies were performed to determine the capacity of the prepared materials for crude oil adsorption. Langmuir and Freundlich adsorption models were used to describe the experimental isotherms. The reliability of the data was examined and evaluated via application of response surface methodology program. The results showed that oil adsorption followed a pseudo-second-order kinetic model and fitted well with Langmuir model with a maximum adsorption capacity of 10.989 and 12.786 g/g for Str and (Str-co-Benz), respectively. Overall, the modified wheat husk is an effective platform for removing oil from marine ecosystems due to low cost, biodegradability, simple synthesis and fast removal. Moreover, the resulted solid can be used as a fuel in some industrial processes such as steam boilers and brick production incinerators.

## Introduction

Environmental pollution is one of the major concerns that has gained significant importance in the past few decades, and associated with health issues including the spread of various diseases, such as typhoid, cholera, cancer and asthma^1^. Anthropogenic activities have been resulted in environmental pollution in all multimedia (air, water and soil). It is estimated that 24% of the global burden of disease and 23% of deaths are due to environmental factors^2^. Environmental pollutants can be categorized into physical, biological and chemical (inorganic and organic)^3^. Physical pollution such as radiation may cause birth defects, burns, some type of leukemia, miscarriages, tumors, cancer of one or more organs and fertility problems^4^. Viruses, bacteria, and/or several forms of pathogens constitute the biological pollutants whereas, inorganic pollutants are concerned with the potentially toxic elements such as mercury, lead, and cadmium. Organic pollutants include the domestic, agricultural, and industrial waste that adversely harm the life and health of animals and human beings^5^. Oil is the most dominant resource of energy as well as an important source of raw materials for synthetic polymers and chemicals all over the world^6^. Oil spills are one of the most catastrophic environmental problems that adversely affect the entire ecosystem and inflict the economy of countries^7^. Over 100 million tons of oil/day are transited by sea, with more than 4000 tankers comprising over 400 million tons’ deadweight on the high seas, putting the marine lives and ecosystem at a dire risk^8^. Due to natural disasters, oil tanker collisions, explosion/fire, hull damage or grounding, pipeline failures, storage tank failures, wells’ collapse, and wars oil spills over marine water or land^9^. Oil type, sea conditions & weather, season, duration and amount spilt, physical, economic, and biological characteristics of spill location, the size of the area oil spreads over, and the effectiveness of the clean-up are among the various parameters that determine how much an oil spill and its response will cost^10^. Oil can severely impact all living organisms by limiting the amount of oxygen reached from the surface and the presence of highly toxic components. Moreover, oil contaminants can directly pollute human food sources, deprive animals and plants of healthy food, affect the food chain, and waste energy resources^11^. Therefore, developing new materials for fast and effective removal of oil spills is mandatory to prohibit the adverse impacts on ecosystems and human health. In this regard, various technologies have been used for the cleanup of oil spills, including conventional physical, mechanical (skimmers, sorbent, and booms), chemical (dispersant and solidifiers), thermal and bioremediation techniques^12^. However, such methods suffer from low separation efficiency, high cost, low stability in strong winds and currents, long time, alteration in aquatic plants and animals and generation of secondary pollutants^11^. Adsorption is one of the most effective methods for oil spill control because of its highly efficient, low cost, simplicity, easy, fast operation, retention over time, and reusability^13^. Many chemically synthetic sorbents, as melamine sponges, polyethylene, and polypropylene have been used due to their high sorption efficiency however, the high-cost, complexity in synthetic processes and non-biodegradability are the drawback that prevent their sustainable application^14^. Thus, searching for new economic, eco-friendly recyclable sorbents with a hydrophobic/oleophilic nature is highly important in oil spill treatment. Ridding the environment from oil contaminants using cellulosic wastes, e.g., rice husk, sugarcane bagasse, and paper wastes, in an eco-friendly and cost-effective way has gained significant attention in the last years. These sorbents can be either used as received or formed into sheets, booms, pads, filters, and fiber assemblies^15^. The numerous advantages of cellulosic wastes (biodegradability, low density, renewability, low cost and non-toxicity) increased the tendency for use instead of synthetic fibers in the environment and industrial development^16^. Moreover, the resulted solid waste can be used in various applications e.g. as fuel in some industrial processes such as steam boilers and brick production incinerators. Various methods (chemical, physical, and functional nanoparticle decoration) have been used to modify the fiber surface, improve fire resistance, adhesion, and hydrophobic properties, and change inexpensive fibers into expensive material^17^. Physical modification involve mechanical pressing or grinding and thermal bonding which have no impact on hydrophobicity improvement of the natural sorbent. In addition, nanoparticle decoration suffers from low capacity and complex preparation process^7^. Chemical modification was carried out via alkylation, hydroxymethylation, esterifcation, acylation and other chemical reactions to remove the weak constituents (hemicelluloses and lignin) and hence improve both the mechanical and adsorption performance^16^. Esterification through grafting polymer onto the surface of the nature adsorbent is a very simple and short time approach to enhance the oil adsorption capacity^7^. To the best of our knowledge, the use of benzoyl chloride for wheat straw modification is not adopted. Thus, the main aim of the present study was to fabricate new eco-friendly, hydrophobic, adsorbent materials based on cellulosic waste through esterification process by grafting wheat straw with benzoyl chloride to increase the affinity for crude oils. The prepared sorbent was characterized and applied for removal of oil spills from oil/seawater system. The results of this study may help decision-makers to develop a long-term strategy to protect the environment from further deterioration and achieve environmental restoration and sustainability.

## Experimental

### Materials

Wheat straw (Str) was collected as agricultural waste sources in Alexandria (Egypt), Sodium hydroxide (99%), and ethanol (99%), were brought from El-Nasr Company (Alexandria). Benzoyl chloride was used as received from Sigma Aldrich. All chemicals were used without further purification. The oil used in this work included diesel motor oil (DO) from Petrol station. The density of DO at room temperature was 0.804 g/l. Real sea water samples were used throughout the whole work to constitutes the oil/water system (see Supplementary Table S1).

### Methods

#### Activation of wheat straw

Wheat straw (agriculture waste) was washed several times with distilled water to remove dust and impurities before filtration and immersed in 5% NaOH for 3 h at room temperature with mechanical stirring. Pure straw was obtained after washing the collected residues several times with water to neutrality and drying at 40 °C^16^.

#### Preparation of wheat straw benzoyl chloride derivative

**Figure 1:**
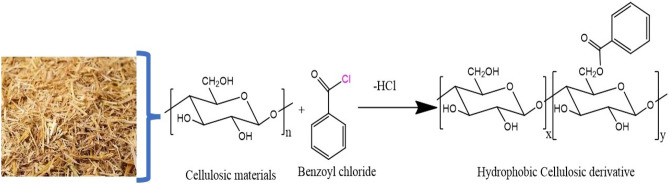
Schematic diagram for the synthesis of (Str-co-Benz).

5 g of the activated wheat straw (Str) was dispersed in 80 ml DMSO in 500 ml boiling flask at room temperature. Then, 4.06 g of benzoyl chloride was added to the flask connected to a condensation column. The temperature of the mixture was raised to 80 °C for 12 h. Subsequently, the flask was cooled and 200.0 ml acetone was added. The wheat straw benzoate ester (Str-co-Benz) derivative was washed twice with acetone on a sintered glass filter and dried under reduced pressure (Fig. 1)^18^.

### Physico-chemical characterization

The chemical structure and the main functional groups of the wheat straw (Str) and the prepared esterified (Str-co-Benz) derivative were investigated using a Fourier Transform Infrared Spectrophotometer (Shimadzu FTIR-8400 S, Japan). Their thermal stability was evaluated using a Thermal Gravimetric Analyzer (Shimadzu TGA-50, Japan) and a Differential Scanning Calorimeter (Shimadzu DSC-60-A, Japan). A Scanning Electron Microscope (SEM; Joel Jsm 6360LA, Japan) was also used to examine the morphological structures of both Str and (Str-co-Benz).

### Uptake in single oil and water systems

For studying the single water and oil uptake behavior of both Str and (Str-co-Benz) grafted copolymers, 0.2 g of each sample was immersed in 50 ml of liquid (water or diesel motor oil) under a constant shaking rate (100 rpm) for 2 h. The swollen samples were separated, and the excess adherent liquid was removed using filter paper, followed by weighing in a closed electronic balance. The following equation can express the liquid uptake (LU)^16^:

1 $$\mathrm{LU }\left(\mathrm{\%}\right)=\left(\frac{\mathrm{Ws}-\mathrm{W}0}{\mathrm{W}0}\right)\times 100$$

where; Ws is the weight of the swollen sample, and W_0_ is the initial dry weight of the sample. The determination of oil or water sorption capacity (Q_O_ or Q_W_; g oil or water per g of sorbent) was carried out by weighing the samples before and after the sorption. The sorption capacity in oil/water system process was conducted according to Keshawy and Farag^12^.

### Batch oil adsorption experiments

The oil adsorption procedure followed the Standard Test Method (ASTM F726-99) for adsorbent performance. Various amounts of oil (1–10 g) were poured into a 250 ml beaker containing 100 ml of natural seawater, followed by varying amounts of adsorbent (0.1–1.5 g) spread on the oil–water surface at different shaking rates (50–250 rpm) for an available contact time (5–90 min). Finally, oil adsorbed were extracted and weighed using an electronic balance^17^. The percentage of oil adsorption was calculated using Eq. (1).

### Response surface methodology (Box–Behnken design; BPD)

The Response Surface Methodology (RSM) is a set of statistical and mathematical tool for designing experiments and optimizing the effect process variables^19^. The correlation of three independent variables (i.e. oil concentration, *X*_*1*_; sorbent dosage, *X*_*2*_; and time, *X*_*3*_) and dependent variable (oil removal capacity, *Y*) was investigated through BPD at three different levels denominated as (− 1, 0, + 1). Two-level factorials can be transformed into BPDs by adding a small number of extra points to evaluate curvature and interaction effects. The layouts can be viewed as three-level partial factorials. A 2^ k^-factorial BPD was performed to build a total of 20 experiments with eight cube points plus six center points and six axial points (see Supplementary Tables S2, S3) for optimization of the three variables that exhibited significant effects on oil removal capacity of Str and (Str-co-Benz). To determine how each independent variable affected responses and model quality, analysis of variance (ANOVA) with Fisher’s exact test and p-value was conducted. The peak’s area was analyzed with a second-order polynomial equation, and the data were fit with a multiple regression model. The statistical relationship between the independent variables and the oil removal capacity (Y) is shown by the quadratic polynomial equation below^20^:

2 $${Y=\beta }_{0}+\sum_{i}{\beta }_{i}{X}_{i}+\sum_{ii}{\beta }_{ii}{X}_{i}^{2}+\sum_{ij}{\beta }_{ij}{X}_{i}{X}_{j}$$

where, *Y* is the predicted response (oil removal capacity g g^-1^); *β*_*0*_ is the model intercept; *X*_*i*_ and *X*_*j*_ are the independent variables, *β*_*i*_ is linear coefficients; *β*_*ij*_ is cross-product coefficients; *β*_*ii*_ is the quadratic coefficients. Multiple determination coefficients (R^2^) and the lack-of-fit value may be used to evaluate how well the model fits the data. With the help of the STATISTICA programme, three-dimensional response surface plots were constructed to analyze the relationship and identify the interactions among the independent and dependent variables.

## Results and discussion

### Characterization

**Figure 2:**
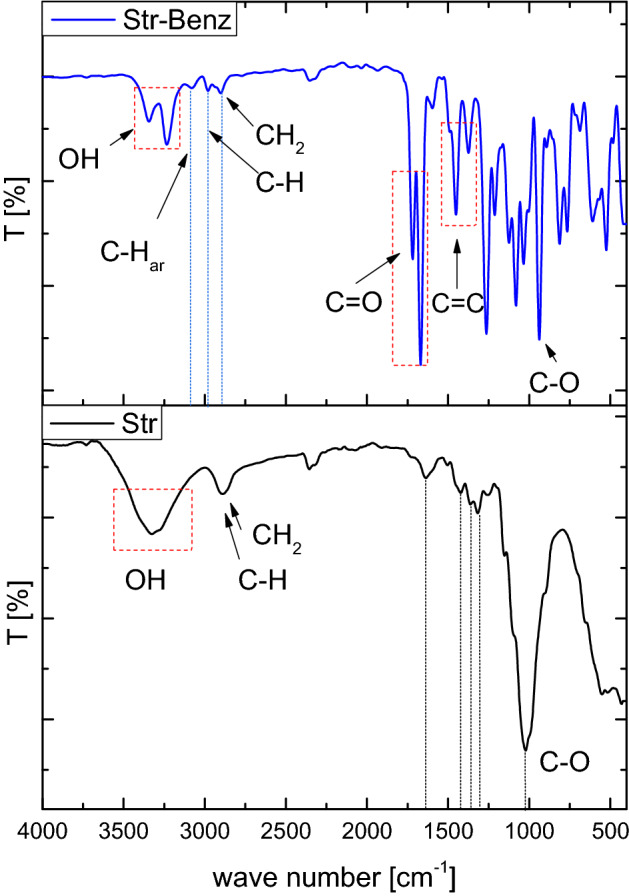
FTIR of wheat straw (Str) and its aromatic derivative (Str-co-Benz).

The FTIR spectra of Str and (Str-co-Benz) was represented in Fig. 2. The broadband between 3100 and 3600 cm^−1^ refers to the stretching vibration of cellulosic hydroxyl groups of Str., bands at 2880–2950 cm^−1^ are related to stretching vibration of the C–H bond and the methylene group –CH_2_. Where, the bands at 1638 cm^−1^ and 1423 cm^−1^ are characteristic for glucopyranose ring of the repeating unit. The weak bands at 1358.9 and 1025 cm^−1^ may be attributed to C–C–H, O–C–H deformation and C–O stretching vibrations. The esterification of wheat straw with benzoyl groups demonstrates a significant variation in the IR spectrum, the vibration bands at 3355 and 3238 cm^−1^ changed to sharp peaks. In addition to the band of C-H at 2991 cm^−1^ and the methylene group at 2894 cm^−1^, a new peak was observed at 3076 that may be due to the aromatic C-H of the benzoyl group. The bands at 1712 and 1456 cm^-1^ may be due to C = O in the ester group and C = C in the benzene ring, respectively.

The TGA curves of Str and (Str-co-Benz) are shown in Supplementary Fig. S1. Three consecutive weight decrease phases were observed, the first step of weight loss involved 7.54% for Str and 10.16% for (Str-co-Benz) from their starting weight, which was most likely owing to the polymer’s elevation of pipe and inter-molecular water molecules. The additional aromatic groups on cellulose chains by esterification were responsible for the increase in intermolecular water in Str as it increases the structure porosity, causing pseudo hydrophilicity. The second weight loss was caused by the complex disintegration processes, which included saccharide rings and macromolecule chains of cellulosic structure of Str and (Str-co-Benz). Raw wheat straw decomposes quickly, with a magnitude of 60.34%, but the (Str-co-Benz) derivative decomposes slowly, with a magnitude of 49.35%. The esterified derivative then degrades faster than neat straw at higher temperatures, with a weight loss of roughly 38.68%. These findings suggested that aromatic groups protect against heat degradation^21^.

DSC experiments completed the thermal characterization, the results of which are shown in Supplementary Fig. S2. The acquired results are in good agreement with the TGA findings. There were some differences in the number of exothermic/endothermic peaks found between constituents and their placements. The thermogram revealed an early endothermic peak for (Str-co-Benz) as a result of moisture content release in the membrane. The ability of (Str-co-Benz) to hold more moisture explains the rise in the endothermic peak over Str. Thermal breakdown of the cellulose chain was attributed to the exothermic peak.

SEM micrographs of Str and (Str-co-Benz) are shown in Supplementary Fig. S3. The roughness of tested surfaces increased with the functionalization of straw with an aromatic ring, as observed in the micrographs. This phenomenon is caused by heterogeneous molecules between the polymeric cellulosic chains, as well as the immobilization of the phenolic group with glucose hydroxyl groups on the repeating polysaccharide, which distorts the internal order of the chains and influences the polymer crystal structure.

### Factors influencing oil adsorption performance using batch technique

The effect of sorbent dosage (g), contact time (min), crude oil initial concentration (g/l), and agitation speed on the sorption of diesel oil (mixed with water) onto the prepared Str and (Str-co-Benz) were examined in order to define the ideal operational parameters controlling the oil adsorption process.

#### Effect of sorbent dosage on oil removal

**Figure 3:**
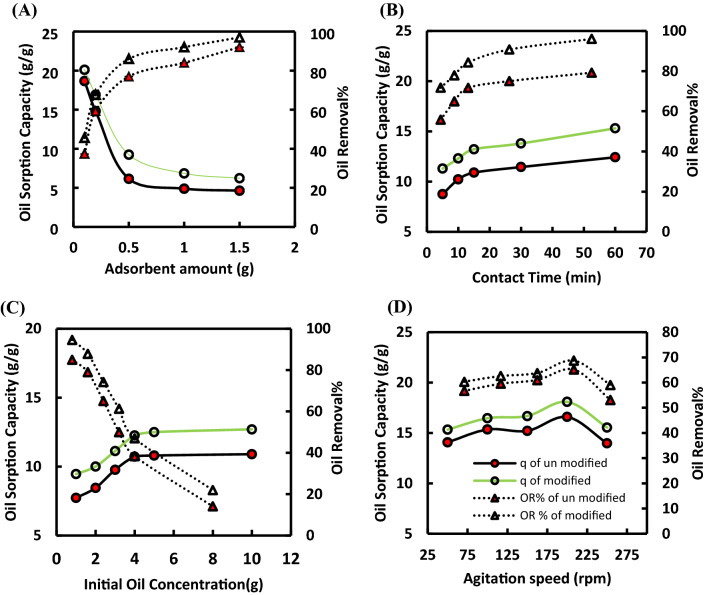
Effect of various parameters on the uptake of crude oil onto Str and (Str-co-Benz) (**A**) adsorbent amount, (**B**) contact time, (**C**) initial oil concentration, and (**D**) agitation speed.

The impact of the adsorbent weight on oil spill cleanup procedures is critical through the large-scale application. The effect of the prepared sorbent dose (0.1–1.5 g) on the percent removal and sorption oil capacity was studied with an initial oil concentration of 5 g/100 ml at 25 °C and 250 rpm. As clear from Fig. 3A, there is an increase in crude oil removal percentage with increasing adsorbent dose and this may be due to increase in the number of active sites available for oil uptake which tend to become unsaturated when compared to lower doses^22^. A remarkable decrease in sorption capacity with increasing sorbent dose was observed and this may be attributed to the residual unsaturated active sites during the process and the under-utilization of active sites caused by sorbent particle aggregation, which is a common phenomenon with clay minerals^23^. The aggregation of the sorbent led to decrease in total surface area and blocking of diffusional holes^24^. Our results agreed with that reported by Omer et al. for the uptake of oil spill by nonanyl chitosan-poly (butyl acrylate) grafted copolymer^25^. The same trend was also recorded for the removal of the crude oil spill by the chitosan and chitosan Schiff base derivatives^26^.

#### Effect of contact time on oil removal

The effect of contact time on oil uptake by Str and (Str-co-Benz) was optimized to determine the equilibrium time for maximum oil uptake and the kinetics of oil adsorption process onto the as-prepared adsorbents. Figure 3B illustrated that increasing the contact time from 5 to 60 min led to increment of the oil adsorption capacity from 8.8 to 12.4 g/g and 11.3 to 15.30 g/g for Str and (Str-co-Benz), respectively as more active sites are available during the first stage of adsorption. Thus, the diffusion of oil molecules onto the external surface of the prepared materials, followed by pore diffusion into the intra-particle matrix until reaching equilibrium. With the passage of time, the adsorbed oil blocks the sites on the adsorptive material’s external surface, preventing the remaining oil from diffusing to the active sites deep within the internal layers^27^. The decrease of adsorbed oil amount after equilibrium may also be due to two phases' opposing sorption mechanisms^28^. The obtained results were consistent with that reported for removal of oil by enzymatic grafting of octadecylamine on corn stalk pith by laccase-TEMPO (2,2,6,6-tetramethylpiperidine-1-oxyl)^29^. The similar behaviour was also recorded for the adsorption of oil using magnetic pomelo peel^24^. It was observed that the equilibrium time of this study is shorter than that reported by Tung et al.^30^.

#### Effect of the initial amount of crude oil on adsorption capacity

The initial concentration of adsorbate is one of the critical factors that affect the adsorption mechanism and controls the overall kinetic coefficient^31^, as the gradient between the solution and adsorbent active sites facilitates particles diffusion through the exterior and interior particle's surface porosity network^32^. The effect of the initial oil amount (1–10 g/100 ml) on adsorption capacity and removal percentage (R%) was investigated. A concentration declivity between the oil–water liquid and the solid adsorbent phase creates a driving force required to overcome all oil resistances between the aqueous and solid phases. Moreover, more oil molecules are available to the adsorbent sites, which enhances the sorption capacity. This is confirmed from Fig. 3C as the oil sorption capacity increases from 7.73 to 10.9 g/g and 9.46 to 12.7 g/g for Str and (Str-co-Benz) when the crude oil concentration raised from 1 up to 4 g/100 ml. In addition, the high adsorption rate and proper utilization of available active sites were taken place with increasing initial crude oil concentration due to the available of active saturation surface sites on the adsorbents^33^. In contrast, the percentage of oil uptake decreased from 85 to 14.10% and 94.60 to 22% for Str and (Str-co-Benz), when the crude oil concentration raised from 1 up to 4 g/100 ml, due to the fact that the adsorbed oil particles eventually begins to clog the adsorbent pores close to the outer surface, preventing oil from diffusing to the active sites buried deep within the interior surface^34^. The obtained results agreed with that recorded for uptake of oil spill by agricultural wastes^35^ and the modified raw flax fiber^16^.

#### Effect of the agitation speed

To simulate weather and wave motions in seawater, it is critical to consider the effect of agitation speed on the oil adsorption process as the rate of mixing is a key component that affects both the formation of the exterior boundary layer and the distribution of oil in the bulk solution^28^. According to the obtained results (Fig. 3D), increasing the shaking speed from 50 to 200 rpm led to increase the crude oil sorption capacity from 14.07 to 16.59, and 15.43 to 18.09 g/g for Str and (Str-co-Benz), as increasing the agitation speed may improve oil dispersal onto the prepared adsorbents’ outer layer^25^. This outcome confirms the impact of external diffusion on the sorption kinetics of the oil adsorption process^36^. On the other hand, a higher agitation speed of more than 200 rpm results in a decrease in oil sorption capacity, which might be due to improving the oil–water emulsion process and reducing the attraction forces between the prepared adsorbent material and oil molecules, thereby promoting the oil desorption process^26^. This tendency for the oil desorption process could be attributed to the high rate of agitation that needed more energy input and resulted in a strong shear force that fractured the bonds between the oil molecules and the adsorbent material^37^. Similar trend was obtained with that reported for uptake of oil spill by agricultural wastes^35^.

### Adsorption isotherms

Langmuir, Freundlich, and Temkin isotherms are the most commonly reported adsorption models for describing the equilibrium between adsorbent and adsorbate^28^. The Langmuir isotherm is based on the assumption that the surface adsorption process displays as a monolayer onto the adsorbent material and denotes the distribution of adsorbate between the solid and liquid phases at equilibrium^38^. The Freundlich isotherm proposed that the adsorbent surface is heterogeneous^39^. While, Temkin suggested that the adsorption isotherm is defined by a homogeneous binding energies distribution with a specified maximum binding energy by reflecting the effects of indirect interactions between the adsorbent and adsorbate^40^. The linear forms of Langmuir, Freundlich, and Temkin isotherm are expressed in the following Equations^28^:

3 $$\frac{C_e}{qe } =\frac{1}{qmKl} + \frac{{C}_{e}}{qm}$$

4 $$log\, {\mathrm{q}}_{\mathrm{e}}={\mathrm{log\,k}}_{\mathrm{f}}+ \frac{1}{{\mathrm{n}}_{\mathrm{f}}}\mathrm{ log\,}{\mathrm{C}}_{\mathrm{e}}$$

5 $${\mathrm{q}}_{\mathrm{e}}=Btln \,KT+Bt\,{\mathrm{ln\,C}}_{\mathrm{e}}$$

where *q*_max_ (g/g), *C*_*e*_ (g/L), and *K*_*L*_ (L/g) are the maximum adsorption capacity for monolayer coverage, the crude oil concentration at equilibrium, and the Langmuir isotherm constant related to the binding site affinity, respectively, 1/*n*_*f*_ and *K*_*f*_ are the Freundlich constants represent adsorption intensity and capacity, *B* (J/mol) is the adsorption heat, and *KT* (L/g) is the highest binding energy of the prepared material and crude oil. The values of the isotherm model's parameters are not only indicators of the adsorbent performance efficiency, but they also enable understanding the adsorption mechanism of crude oil toward the modified and unmodified materials surface. The dimensionless separation factor (${\mathrm{R}}_{\mathrm{L}}=\frac{1}{1+{\mathrm{K}}_{\mathrm{L}} {\mathrm{C}}_{0}}$) was used to predict the adsorption phenomenons feasibility and isotherms nature^29^, which may be irreversible (R_L_ = 0), favorable (0 < R_L_ < 1), linear (R_L_ = 1) or reversible (R_L_ > 1). By fitting the Langmuir model, the R_L_ values were 0.0128 and 0.0104 for unmodified and modified materials, respectively, which indicates a favorable adsorption process with good correlation coefficient (R^2^ = 0.99 and 0.999) in contrast to the values obtained from Freundlich (0.95 and 0.98) and Temkin (0.899 and 0.94) isotherms models, implying a monolayer adsorption coverage with homogeneous adsorption energy^30^.

**Figure 4:**
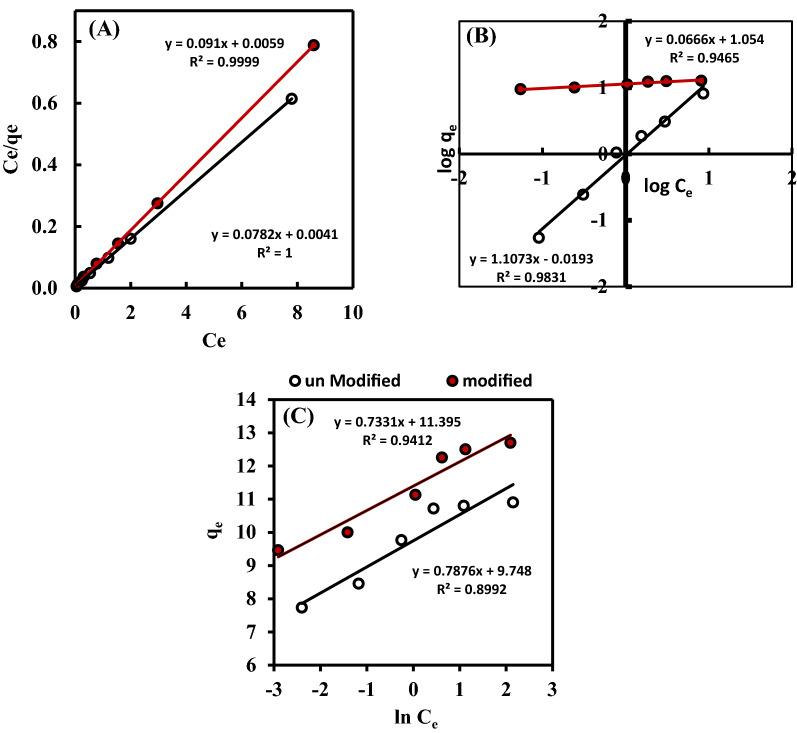
(**A**) Langmuir (**B**) Freundlich, and (**C**) Temkin adsorption isotherm models for the uptake of crude oil onto Str and (Str-co-Benz).

The Freundlich isotherm is an empirical model that depends on an exponential distribution between the adsorption sites and energies and describes the amount of adsorbed crude oil per unit mass of the adsorbent. From the Freundlich isotherm model parameters, the value of 1/n demonstrates the nature of the adsorption process, as it will be heterogeneous adsorption if 1/n < 1 and cooperative adsorption if 1/n > 1^10^. Besides, the *B*_*T*_ parameter obtained from Temkin plot has positive and high values, indicating a more significant interaction between the adsorbent and the adsorbate (see Supplementary Table S4 and Fig. 4). From the obtained results it is found that Langmuir model is more effective at predicting the intensity and adsorption capacity of Str and (Str-co-Benz), compared to the Freundlich model confirming monolayer chemisorption of oil particles. The obtained data is similar to that recorded by Peng et al.^29^ and Kaur & Sodhi^35^.

### Adsorption kinetics

**Figure 5:**
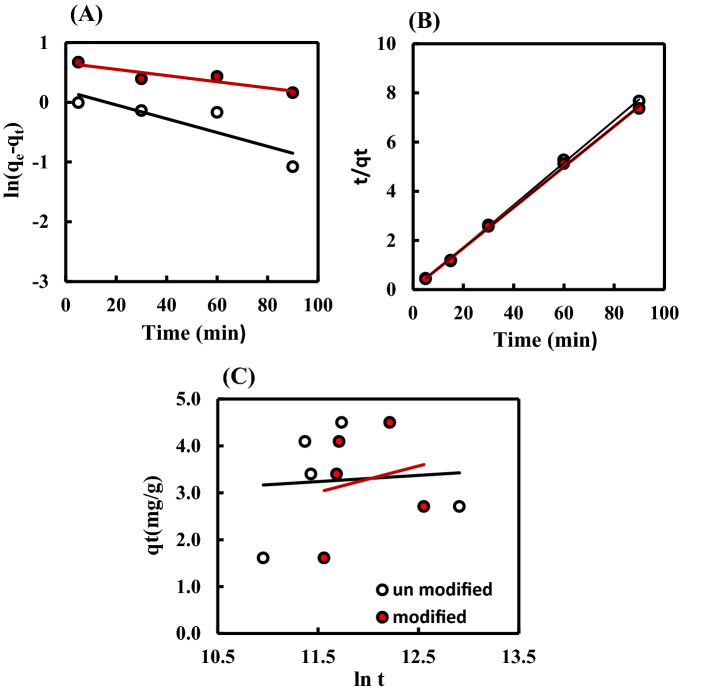
Kinetics models for the adsorption of the crude oil onto the prepared Str and (Str-co-Benz), (**A**) pseudo-first-order, (**B**) pseudo-second-order, and (**C**) simple Elovich kinetics models.

The kinetics of crude oil adsorption onto Str and (Str-co-Benz) was investigated as a function of contact time. The experimental data were examined using the pseudo-first-order, pseudo-second-order, and Elovich kinetics models (see Supplementary Table S5 and Fig. 5) to demonstrate the reaction mechanism of the adsorption process^31^. A correlation between the experimental (q_exp_) and calculated (q_cal_) values of oil sorption capacity was displayed in Supplementary Table S6. The q_cal_ values of the pseudo-second-order kinetic model were very close to the q_exp_ values, whereas the pseudo-first-order exhibited a significant difference. Furthermore, the correlation coefficient (R^2^) values are close to 1 for both Str and (Str-co-Benz) revealing that the pseudo-second-order model is more appropriate for describing the obtained data, and the adsorption mechanism is mainly chemisorption^33^. As a result, the Elovich model should be applied^34^ to assess the contribution of other forces (in addition to Vander Waals forces) participating in the adsorption process. Moreover, this model assumed that the actual sorbent surfaces have heterogeneous energies^36^. The same trend was obtained by Mahmoud^16^ and Peng et al.^29^ as they demonstrated that the oil sorption via chemical bonding was usually best described with the pseudo-second kinetic model.

### Response surface methodology (RSM)

RSM is a reliable, robust, and efficient mathematical technique that incorporates statistical experimental designs and multiple regression analysis for finding the optimal formulation under a set of restricted equations^41^. The design matrix, experimental and predicted results, the codes and actual values of the selected variables are shown in Supplementary Table S2. It is clear that the three independent variables greatly affected the value of oil removal capacity (g/g). For the (Str-co-Benz), the highest oil removal capacity of ~ 17.26 g g^−1^ (predicted to be 16.39 g g^−1^) was observed at the factorial level concentrations of the chosen variables in experimental trial number 4. While, the lowest oil removal capacity of 8.568 g g^−1^ (predicted to be 10.37 g g^−1^) was recorded in trial 19 when all predictors were held at their center points (zero levels) except oil concentration which was at the lowest or axial level (− 1) with a concentration of 1.5 g. This confirms the integrated effect of adsorbent dose, time and initial oil concentration on capacity value which were discussed in Section “Factors influencing oil adsorption performance using batch technique”. On the other hand, the Str obtained results showed that the highest oil removal capacity of ~ 16.27 g g^−1^ (predicted to be 15.07 g g^−1^) was obtained at factorial level concentrations of the chosen variables in experimental trial number 20, where, all selected variables fall in the highest values. In contrast, the lowest oil removal capacity of 4.32 g g^−1^ (predicted to be 5.73 g g^−1^) was observed in trial 11 when all predictors were held at their highest points (+ 1 levels) except time which was provided at its lowest point (− 1 level) with time duration of 36 min, this illustrates and affirms the effect of contact time on the adsorption process as mentioned in Section “Effect of contact time on oil removal”.

### Multiple regression analysis and ANOVA

The mathematical operators of multiple regression statistical analysis and ANOVA calculations used to analyze and interpret the BPD experimental data are summarized in Supplementary Tables S7 and S8. ANOVA’s principal purpose is to compare procedure variability with residual error and to determine the relevance of the regression model. Only if the residues (square of the difference between the predicted and experimental response) have a normal distribution and homogeneity, the regression analysis is valid^42^. The models were characterized by Fisher’s *F*-test of 7.07 and 4.5, with the low probability* p* of 0.0025 and 0.013 for Str and (Str-co-Benz), indicating that the chosen model is very meaningful, as clear from the ratio of mean square regression and mean square residual. *p*-values, *F*-values, and *t* tests were used to verify the significance of each variable, and to recognize the reciprocal interaction dynamics between variables under consideration. In addition, depending on the sign of the coefficients, the interrelation between two variables might have a positive or negative effect on the response, where a positive sign indicates a synergistic or complementary influence of two variables on the response, and a negative sign denotes an antagonistic effect. Regression coefficients were calculated to find the best fit for the second-order polynomial equation. Oil removal capacity (*Y*) using the (Str-co-Benz) is expressed as a regression equation:

6 $${Y}_{capacity }=12.48+1.93{X}_{1}-0.732{X}_{2}+2.182{X}_{3}+1.35{X}_{1}{X}_{2}+0.305{X}_{1}{X}_{3}-0.111{X}_{2}{X}_{3}-0.183{X}_{1}^{2}+0.311{X}_{2}^{2}-0132{X}_{3}^{2}.$$

However, the capability (Y) to remove oil using the Str is represented as:

7 $${Y}_{capacity }=9.55+0.260{X}_{1}-0.624{X}_{2}+2.250{X}_{3}+0.140{X}_{1}{X}_{2}+0.496{X}_{1}{X}_{3}+1.922{X}_{2}{X}_{3}-0.594{X}_{1}^{2}+0.09{X}_{2}^{2}+1.583{X}_{3}^{2},$$

where *Y* is the predicted response (Oil removal capacity), and *X*_*1*_, *X*_*2*_, and *X*_*3*_ are the coded levels of the independent variables of oil conc., sorbent dosage, and time, respectively.

### Contour and three-dimensional (3D) plots

**Figure 6:**
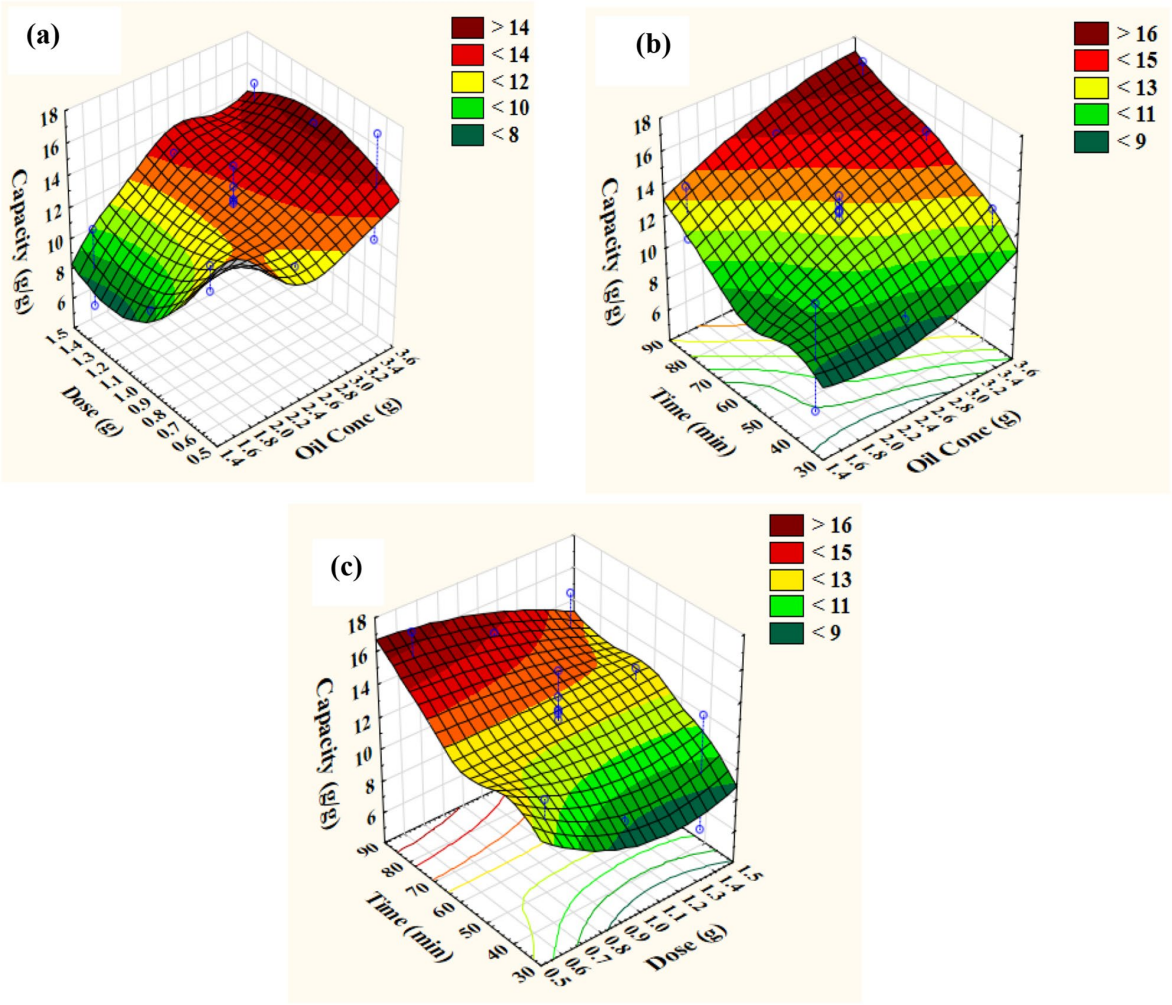
3D response surface representing oil removal capacity by modified wheat straw as affected by incubation conditions: (**a**) Interaction between dose and oil concentration, (**b**) Interaction between time and oil concentration, (**c**) Interaction between time and dose.

**Figure 7:**
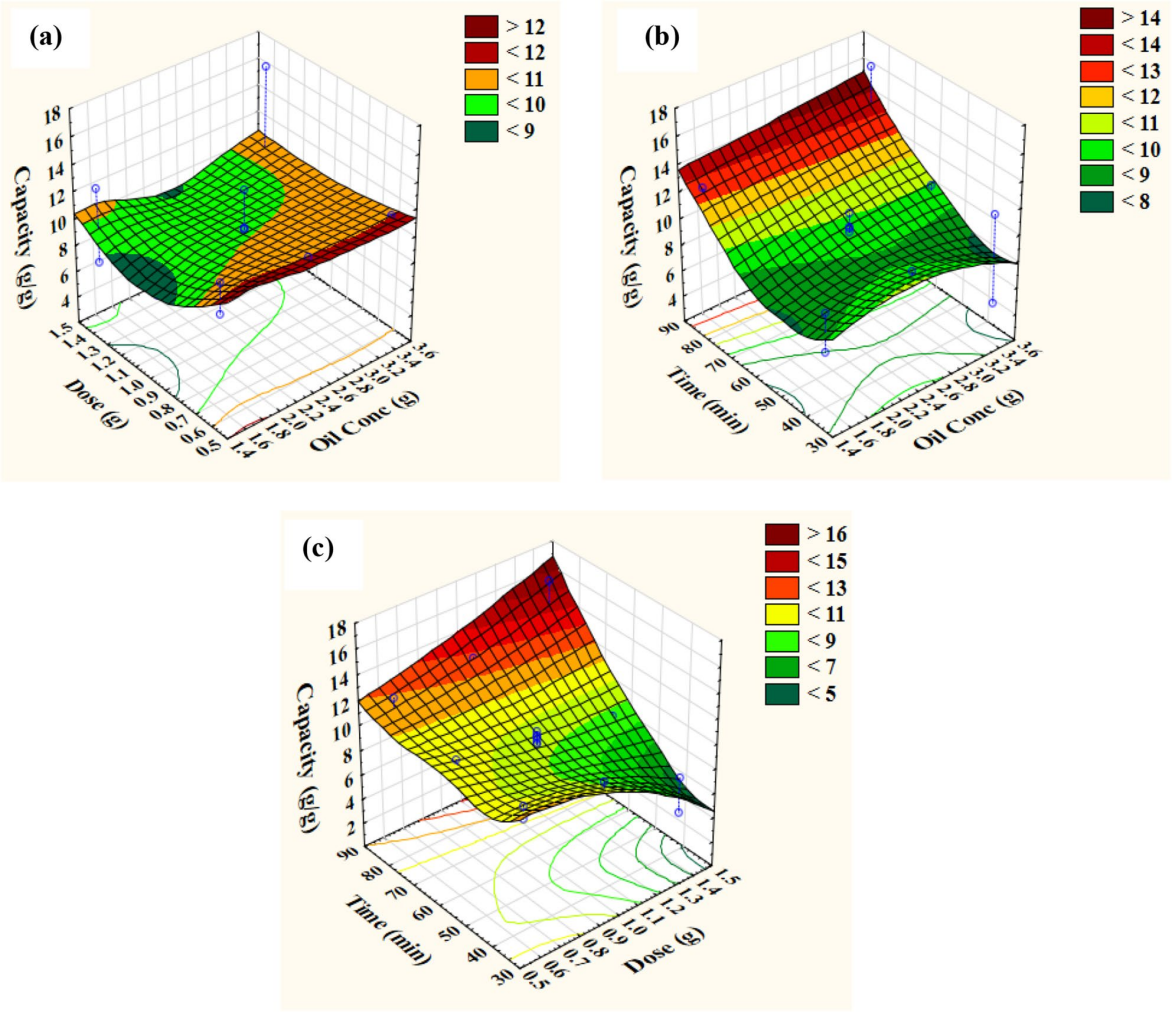
3D response surface representing oil removal capacity by unmodified wheat straw as affected by incubation conditions: (**a**) Interaction between dose and oil concentration, (**b**) Interaction between time and oil concentration, (**c**) Interaction between time and dose.

To investigate the performance of a design across the experimental region, graphical techniques have been developed. A three-dimensional response surface and two-dimensional contour plots can be used to visualize the effects of interactions between variables under investigation on responses based on the regression model developed^43^. Three-dimensional plots for the significant pair-wise combinations of the three variables (X_1_X_2_, X_1_X_3_, and X_2_X_3_) were constructed by plotting response (oil removal capacity) on the z-axis against two independent factors while leaving other variables at zero levels (center points), as shown in Figs. 6 and 7. The combined effects of oil concentration and sorbent dosage on oil removal capacity by using modified and unmodified wheat straw were shown in a three-dimensional surface map (Figs. 6a, 7a). The data revealed that with increasing oil concentrations, the oil removal capacity increased until it reached its optimum, but increase sorbent dosage led to low oil removal capacity due to the aggregation of the sorbent which led to decrease in total surface area and blocking of diffusional holes which ascertained the results obtained in Section “Effect of sorbent dosage on oil removal”. The maximum capacity (Figs. 6b and 7b) was achieved at high level of both oil concentration (*X*_*1*_) and time (*X*_*3*_). Maintaining oil concentration at zero levels with increase contact time beyond the center point led to oil removal capacity enhancement (sorbent dosage at the lower point); whereas increasing the concentrations above the recorded point led to a drop of oil removal capacity as illustrated in Figs. 6c, 7c.

### Comparison of capacity obtained by the present study with other adsorbents

Table 1 displayed the adsorption capacity of Str and (Str-co-Benz) compared to other adsorbents. The data revealed that the proposed materials exhibited good adsorption capacity for diesel oil in comparison with previously reported materials with various advantages, including better surface chemistry, fast removal rates, simple preparation process and it can be used without modification. Even if these natural adsorbents do not perform as well as conventional adsorbents, they are still a good alternative because they are already a waste material. They can be utilized, without turning the adsorbent manufacturing and designing process itself into a pollution source.

**Table 1:** Comparison of oil adsorption capacities of the present study with other oil adsorbing materials.

No	Adsorbent	Capacity (g/g)	Ref
1	Raw, pretreated and acetylated wheat straw	8.19, 15.67 and 24.21	^44^
2	Kenaf core fibers		
	Kenaf sachet	3.6	^45^
	Kenaf powder	1.4	
3	Polystyrene (PS) fibers	20.99	^46^
4	Hexadecyltrimetylammonium bromide modified Nigerian nanoclays		
	OCA	1.98	^47^
	OCB	1.88	
	OCC	1.9	
5	Magnetic pomelo peel (MPP)	27.98	^24^
6	Soil	0.012	^48^
7	Modified natural fibers		
	LCLA and	11.00	^49^
	PFLA	8.9	
8	Magnetic activated carbon nanoparticles	6.5	^50^
9	Str	10.989	Present study
	Str-co-Benz	12.786	

## Conclusion

Oil is the most dominant resource of energy as well as an important source of raw materials for synthetic polymers and chemicals all over the world. Oil spill is one of the major environmental problems with a serious impact on both humans and the ecosystem. The development of hydrophobic sorbents for the removal of spilled oil is very important for ecological preservation and sustainable development. This work demonstrated the use of raw and benzoyl chloride modified wheat straw as low-cost adsorbent material for the removal of oil from water/oil system. An optimization study was conducted at various concentrations of crude oil, adsorbent dose, agitation time, and speed. Oil sorption capacity of 10.989 and 12.786 g/g was obtained for Str and (Str-co-Benz) materials, respectively. The resulted data fitted well with Langmuir isotherm, (R^2^=0.999) conforming mono-layer adsorption of crude oil onto the prepared materials. The kinetic studies revealed that the Pseudo-second-order kinetic models fitted well with the obtained results for the removal of crude oil with highest correlation coefficient (R^2^) confirming a chemisorption process. Moreover, the reliability of obtained results were confirmed by application of RSM mathematical approach through integration effect of the tested variables on adsorption process. These natural adsorbents (as agro-waste material) are still a good alternative to the used conventional adsorbents, as the use of waste materials for waste-to-value purposes has increased the attention of both scientists and environmental organizations. The outcomes of this study may help decision-makers to develop a long-term strategy to protect the environment from further deterioration and achieve environmental restoration. As a future perspective, a special emphasis should be considered for surface modification of agricultural wastes to be used for removal of oil spill at real field applications.

## Supplementary Information

Supplementary Information.

## Data Availability

All data generated or analyzed during this study are included in this manuscript [and its supplementary information file].
