# Supplementary material for: Wheat husk-based sorbent as an economical solution for removal of oil spills from sea water
Source: Sci Rep. 2023 Feb 13;13:2575. doi: 10.1038/s41598-023-29035-8 (PMC9925805; doi:10.1038/s41598-023-29035-8)
Supplement: Supplementary file 1 — Supplementary Information. [file 41598_2023_29035_MOESM1_ESM.docx]

**Wheat Husk-based Sorbent as an Economical Solution for Removal of Oil Spills from Sea Water**

| Parameter | Value ± SD |
| --- | --- |
| pH | 8.3±0.03 |
| Turbidity (NTU) | 8.25±0.2 |
| Conductivity (mmhos/cm) | 56.2± 1.2 |
| Salinity (g/L) | 39.4±0.8 |
| Oil density (g/L) | 0.804±0.007 |

**Table S1.** Characteristics of the seawater sample and motor diesel oil used in the present study.

|  | **Trials** |  | **Variables** | | | | | **Oil removal capacity (g g^-1^ )** | | | |
| --- | --- | --- | --- | --- | --- | --- | --- | --- | --- | --- | --- |
| **Std.Order** |  | **Type** | *X_1_* | | *X_2_* | *X_3_* | | Actual value | | Predicted value | Residual |
| 20 | 1 | Center | 0 | 0 | | 0 | | 12.762 | | 12.48706 | 0.274 |
| 11 | 2 | Axial | 0 | -1 | | 0 | | 11.55 | | 13.53065 | -1.98 |
| 14 | 3 | Axial | 0 | 0 | | 1 | | 15.114 | | 14.53545 | 0.578 |
| 6 | 4 | Factorial | 1 | -1 | | 1 | | 17.265 | | 16.39096 | 0.874 |
| 12 | 5 | Axial | 0 | 1 | | 0 | | 13.38214 | | 12.0658 | 1.316 |
| 10 | 6 | Axial | 1 | 0 | | 0 | | 15.375 | | 14.23728 | 1.137 |
| 3 | 7 | Factorial | -1 | 1 | | -1 | | 5.989286 | | 6.697247 | -0.707 |
| 16 | 8 | Center | 0 | 0 | | 0 | | 11.412 | | 12.48706 | -1.075 |
| 1 | 9 | Factorial | -1 | -1 | | -1 | | 12.715 | | 10.65068 | 2.064 |
| 18 | 10 | Center | 0 | 0 | | 0 | | 12.387 | | 12.48706 | -0.100 |
| 4 | 11 | Factorial | 1 | 1 | | -1 | | 13.17 | | 12.66486 | 0.505 |
| 15 | 12 | Center | 0 | 0 | | 0 | | 13.806 | | 12.48706 | 1.318 |
| 19 | 13 | Center | 0 | 0 | | 0 | | 12.9 | | 12.48706 | 0.412 |
| 5 | 14 | Factorial | -1 | -1 | | 1 | | 14.285 | | 14.62406 | -0.339 |
| 7 | 15 | Factorial | -1 | 1 | | 1 | | 11.00786 | | 10.22313 | 0.784 |
| 13 | 16 | Axial | 0 | 0 | | -1 | | 8.931 | | 10.17385 | -1.242 |
| 17 | 17 | Center | 0 | 0 | | 0 | | 12.984 | | 12.48706 | 0.496 |
| 2 | 18 | Factorial | 1 | -1 | | -1 | | 10.575 | | 11.19365 | -0.618 |
| 9 | 19  2 | Axial | -1 | 0 | | 0 | | 8.568 | | 10.37003 | -1.802 |
| 8 | 20 | Factorial | 1 | 1 | | 1 | | 15.51643 | | 17.41468 | -1.898 |
| **Variables** | | **Code** | **Coded and actual levels** | | | | | | | |  |
|  |  |  | -1 | | | | 0 | | 1 | |  |
| Oil Conc., (g) | | *X_1_* | 1.5 | | | | 2.5 | | 3.5 | |  |
| Dose (g) | | *X_2_* | 0.6 | | | | 1 | | 1.4 | |  |
| Time (min.) | | *X_3_* | 36 | | | | 60 | | 84 | |  |

**Table S2.** Matrix designed for oil removal by (Str-co-Benz) using Box–Behnken design.

|  | **Trials** |  | **Variables** | | | | | **Oil removal capacity (g g^-1^ )** | | | |
| --- | --- | --- | --- | --- | --- | --- | --- | --- | --- | --- | --- |
| **Std.Order** |  | **Type** | *X_1_* | | *X_2_* | *X_3_* | | Actual value | | Predicted value | Residual |
| 20 | 1 | Center | 0 | 0 | | 0 | | 9.83 | | 9.5521 | 0.2778 |
| 11 | 2 | Axial | 0 | -1 | | 0 | | 11.00833 | | 10.267 | 0.7413 |
| 14 | 3 | Axial | 0 | 0 | | 1 | | 12.765 | | 13.386 | -0.6213 |
| 6 | 4 | Factorial | 1 | -1 | | 1 | | 10.9 | | 12.200 | -1.3006 |
| 12 | 5 | Axial | 0 | 1 | | 0 | | 8.614286 | | 9.017 | -0.4035 |
| 10 | 6 | Axial | 1 | 0 | | 0 | | 10.21 | | 9.2178 | 0.9921 |
| 3 | 7 | Factorial | -1 | 1 | | -1 | | 7.146429 | | 5.930 | 1.21621 |
| 16 | 8 | Center | 0 | 0 | | 0 | | 9.335 | | 9.552 | -0.2171 |
| 1 | 9 | Factorial | -1 | -1 | | -1 | | 10.02083 | | 11.304 | -1.284 |
| 18 | 10 | Center | 0 | 0 | | 0 | | 9.62 | | 9.552 | 0.0678 |
| 4 | 11 | Factorial | 1 | 1 | | -1 | | 4.328571 | | 5.738 | -1.4095 |
| 15 | 12 | Center | 0 | 0 | | 0 | | 9.195 | | 9.552 | -0.3571 |
| 19 | 13 | Center | 0 | 0 | | 0 | | 8.7425 | | 9.552 | -0.8096 |
| 5 | 14 | Factorial | -1 | -1 | | 1 | | 12.29167 | | 10.966 | 1.32506 |
| 7 | 15 | Factorial | -1 | 1 | | 1 | | 12.68 | | 13.282 | -0.6027 |
| 13 | 16 | Axial | 0 | 0 | | -1 | | 9.845 | | 8.8859 | 0.9590 |
| 17 | 17 | Center | 0 | 0 | | 0 | | 9.915 | | 9.5521 | 0.3628 |
| 2 | 18 | Factorial | 1 | -1 | | -1 | | 11.07083 | | 10.552 | 0.5183 |
| 9 | 19  2 | Axial | -1 | 0 | | 0 | | 8.0425 | | 8.6969 | -0.6544 |
| 8 | 20 | Factorial | 1 | 1 | | 1 | | 16.27679 | | 15.077 | 1.1996 |
| **Variables** | | **Code** | **Coded and actual levels** | | | | | | | |  |
|  |  |  | -1 | | | | 0 | | 1 | |  |
| Oil Conc., (g) | | *X_1_* | 1.5 | | | | 2.5 | | 3.5 | |  |
| Dose (g) | | *X_2_* | 0.6 | | | | 1 | | 1.4 | |  |
| Time (min.) | | *X_3_* | 36 | | | | 60 | | 84 | |  |

**Table S3.** Matrix designed for oil removal by Str using Box–Behnken design.

| Isotherm parameters | Str | | (Str-co-Benz) |
| --- | --- | --- | --- |
| Langmuir | | | |
| q_m_ (g g^−1^) calculated | 10.989 |  | 12.786 |
| K_L_ (L mg^−1^) | 15.383 |  | 18.878 |
| R^2^ | 0.99 |  | 0.9999 |
| Freundlich | | | |
| K_F_ (mg1^− 1^/n L1/n g^−1^) | 1.038 |  | 11.325 |
| nf | 0.98 |  | 15.02 |
| R^2^ | 0.95 |  | 0.98 |
| Temkin | | | |
| B (L mg^−1^) | 0.788 |  | 0.733 |
| K_T_ (KJ mol^−1^) | 2*10^5 |  | 6*10^6 |
| R^2^ | 0.899 |  | 0.94 |

**Table S4.** Langmuir, Freundlich, and Temkin parameters for adsorption of crude oil (4 g/100 ml) onto 0.1 gm of Str and (Str-co-Benz) at 25 ^°^C.

.

| Adsorption kinetic models | Equation | Parameters |
| --- | --- | --- |
| Pseudo-First-Order | *ln (q_e_ - q_t_) =ln q_e_ - K_1_ t* | qt and q_e_ are the crude oil adsorption capacity at *t* (min) and equilibrium (mg/g), respectively. k_1_ (min^-1^) is the first-order reaction rate constant. |
| Pseudo-Second-Order | $\frac{t}{q_{t}}$*=* $\frac{1}{k^{2}q_{e}^{2}}$ *+*$\frac{t}{q_{e}}$ | q_t_ and q_e_ are the crude oil adsorption capacity at time *t* (min) and equilibrium (mg/g), respectively, and k_2_ is the second-order reaction rate equilibrium constant (g/mg min). |
| Elovich | *q_t_ = ὰ +ß ln t* | ὰ is the initial adsorption rate (mg/g min), and ß is the extent of surface coverage and activation energy for chemisorption (g/mg) |

**Table S**5. Kinetic Models for the uptake of crude oil onto Str and (Str-co-Benz).

| Kinetic model | Kinetic parameters Unmodified | | | Modified |
| --- | --- | --- | --- | --- |
| Pseudo-First-Order | q_e_ (g/g) Calculated | 12.55 | 12.91 | |
|  | q_e_ (g/g) Experimental | 0.525 | 1.036 | |
|  | k_1_ (min^_1^) | 0.0125 | -0.009 | |
|  | R^2^ | 0.11 | 0.636 | |
| Pseudo-Second-Order | q_e_ (g/g) Calculated | 12.55 | 12.91 | |
|  | q_e_ (g/g) Experimental | 11.587 | 12.109 | |
|  | k_2_ (g/mg min) | 0.486 | 0.462 | |
|  | R^2^ | 0.999 | 0.999 | |
| Elovich | ß (g/mg) | 1.879 | 0.846 | |
|  | ὰ (mg/g min) | 8.084 | 10.106 | |
|  | R^2^ | 0.890 | 0.882 | |

**Table S6.** Pseudo-first-order, Pseudo-second–order, and Elovich kinetics models parameters for the adsorption of crude oil (4 g/100 ml) onto 0.1 gm of Str and (Str-co-Benz) at 25 ^°^C.

| Term | Coefficient Estimate | Standard Error | *t*-Stat | *F*-value | *p*-value | Confidence level (%) | Sum of Squares |
| --- | --- | --- | --- | --- | --- | --- | --- |
| Intercept | 12.48706 | 0.5540 | 22.53 |  |  |  |  |
| X_1_ | 1.933629 | 0.5096 | 3.794 | 14.3949 | 0.00352 | 99.64801 | 37.3892 |
| X_2_ | -0.73243 | 0.5096 | -1.437 | 2.0654 | 0.181223 | 81.87771 | 5.364516 |
| X_3_ | 2.1808 | 0.5096 | 4.279 | 18.3103 | 0.001614 | 99.83862 | 47.55889 |
| X_1_*X_2_ | 1.356161 | 0.5698 | 2.380 | 5.6647 | 0.038608 | 96.13916 | 14.71338 |
| X_1_*X_3_ | 0.305982 | 0.5698 | 0.536 | 0.2884 | 0.603011 | 39.69891 | 0.749001 |
| X_2_*X_3_ | -0.11188 | 0.5698 | -0.196 | 0.0385 | 0.848276 | 15.17237 | 0.100128 |
| X_1_*X_1_ | -0.18341 | 0.9718 | -0.188 | 0.0356 | 0.854087 | 14.59131 | 0.092507 |
| X_2_*X_2_ | 0.311162 | 0.9718 | 0.320 | 0.1025 | 0.755427 | 24.45735 | 0.266261 |
| X_3_*X_3_ | -0.13241 | 0.9718 | -0.136 | 0.0186 | 0.894332 | 10.56675 | 0.048213 |
|  |  |  |  |  |  |  |  |
|  | **df** | **SS** | **MS** | ***F*** | **Significance *F*** |  |  |
| **Regression** | 9 | 106.171 | 11.796 | 4.541 | 0.01347 |  |  |
| **Residual** | 10 | 25.973 | 2.5973 |  |  |  |  |
| **Total** | 19 | 132.145 |  |  |  |  |  |
| ***R^2^*** | 0.803 |  |  |  |  |  |  |
| **Adj. *R^2^*** | 0.626 |  |  |  |  |  |  |
|  |  |  |  |  |  |  |  |

**Table S7.** Analysis of variance for the response surface of oil uptake onto (Str-co-Benz) obtained by Box. “(R^2^) is the determination coefficient, Adj R^2^ is the adjusted-R^2^”.

| Term | Coefficient Estimate | Standard Error | *t*-Stat | *F*-value | *p*-value | Confidence level (%) | Sum of Squares |
| --- | --- | --- | --- | --- | --- | --- | --- |
| Intercept | 9.552166 | 0.4225 | 22.605 |  |  |  |  |
| X_1_ | 0.260476 | 0.3886 | 0.6701 | 0.4491 | 0.517946 | 48.20542 | 0.678478 |
| X_2_ | -0.62456 | 0.3886 | -1.606 | 2.5819 | 0.139172 | 86.08279 | 3.900746 |
| X_3_ | 2.250179 | 0.3886 | 5.7891 | 33.5139 | 0.000176 | 99.98245 | 50.63304 |
| X_1_*X_2_ | 0.140074 | 0.4345 | 0.3223 | 0.1039 | 0.753841 | 24.61589 | 0.156967 |
| X_1_*X_3_ | 0.496622 | 0.4345 | 1.1427 | 1.306 | 0.279748 | 72.02521 | 1.973067 |
| X_2_*X_3_ | 1.922723 | 0.4345 | 4.4244 | 19.5756 | 0.001285 | 99.87148 | 29.57492 |
| X_1_*X_1_ | -0.59479 | 0.7412 | -0.8024 | 0.6439 | 0.440939 | 55.90606 | 0.972878 |
| X_2_*X_2_ | 0.090271 | 0.7412 | 0.1217 | 0.0148 | 0.905479 | 9.452125 | 0.022409 |
| X_3_*X_3_ | 1.583961 | 0.7412 | 2.1370 | 4.5668 | 0.058328 | 94.16717 | 6.899565 |
|  |  |  |  |  |  |  |  |
|  | **df** | **SS** | **MS** | ***F*** | **Significance *F*** |  |  |
| **Regression** | 9 | 96.154 | 10.683 | 7.0716 | 0.00259 |  |  |
| **Residual** | 10 | 15.1080 | 1.5108 |  |  |  |  |
| **Total** | 19 | 111.262 |  |  |  |  |  |
| ***R^2^*** | 0.864 |  |  |  |  |  |  |
| **Adj. *R^2^*** | 0.7420 |  |  |  |  |  |  |
|  |  |  |  |  |  |  |  |

**Table S8.** Analysis of variance for the response surface of oil uptake onto Str obtained by Box. “ (R^2^) is the determination coefficient, Adj R^2^ is the adjusted-R^2^”.

**Figure S1.** TGA of Str and its aromatic derivative (Str-co-Benz).

**Figure S2.** DSC of Str and its aromatic derivative (Str-co-Benz).

| Str | 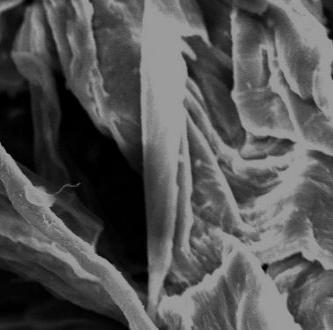 | 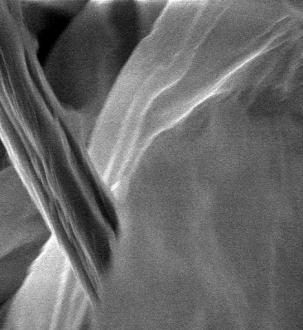 | 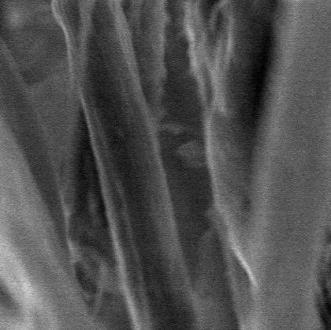 |
| --- | --- | --- | --- |
| Str-co-Benz | 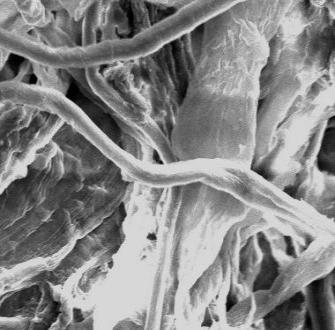 | 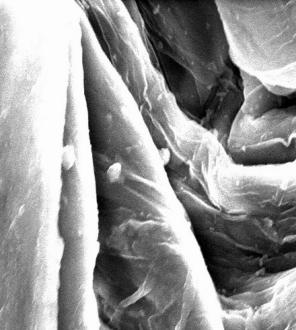 | 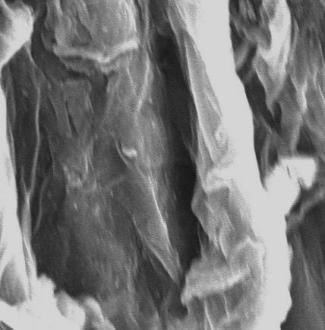 |

**Figure S3.** SEM images of Str and its aromatic derivative (Str-co-Benz).
